# Comparison of risk factors for cardiovascular disease in hemodialysis and peritoneal dialysis patients

**DOI:** 10.6061/clinics/2015(09)01

**Published:** 2015-09

**Authors:** Ozlem Harmankaya, Nilgul Akalin, Hatice Akay, Yildiz Okuturlar, Kayhan Erturk, Hakan Kaptanogullari, Hakan Kocoglu

**Affiliations:** I Bakırköy Dr. Sadi Konuk Teaching Hospital, Division of Nephrology, Istanbul, Turkey; II Biruni University, Department of Dialysis Tecniciant, Istanbul, Turkey

**Keywords:** Cardiovascular Risk Factors, Atherosclerosis, Hemodialysis, Peritoneal Dialysis, Kidney Failure

## Abstract

**OBJECTIVE:**

In this study, we aimed to compare the cardiovascular risk factors that might be associated with inflammation, atherosclerosis and metabolic syndrome between hemodialysis and peritoneal dialysis patients.

**METHODS:**

Fifty hemodialysis and 50 peritoneal dialysis patients who had been receiving dialysis therapy for at least one year were included in the study. Venous blood samples were taken after 12 hours of fasting, and serum glucose, triglyceride, low-density lipoprotein (LDL)-cholesterol, high-density lipoprotein (HDL)-cholesterol, C-reactive protein, fibrinogen and homocysteine levels were measured. The presence of atherosclerotic plaques in the carotid artery was evaluated by carotid Doppler ultrasound. These data were analyzed by Student's t test, the chi-square test and the Mann-Whitney U test, as appropriate.

**RESULTS:**

No difference was found between the hemodialysis (n=50) and peritoneal dialysis (n=50) patient groups regarding mean age, gender distribution, body mass index or dialysis duration (*p=*0.269, 0.683, 0.426, and 0.052, respectively). LDL-cholesterol, fibrinogen and homocysteine levels were significantly higher in peritoneal dialysis patients (*p=*0.006, 0.001, and 0.002, respectively). In patients with diabetes mellitus (n=17) who were undergoing renal replacement therapy, LDL-cholesterol and fibrinogen levels were significantly higher than in patients without diabetes mellitus who were undergoing renal replacement therapy (*p=*0.001 and 0.004, respectively).

**CONCLUSION:**

In our study, cardiovascular risk factors (especially LDL-cholesterol) were more frequent in peritoneal dialysis patients than in hemodialysis patients.

## INTRODUCTION

The incidence of cardiovascular disease is approximately 25-65% in patients with chronic kidney disease, and it may increase 20- to 40-fold in dialysis patients compared with the healthy population 1. Several factors increase the risk of cardiovascular disease in dialysis patients. Modifiable factors such as anemia, mineral metabolism disorders, uncontrolled arterial blood pressure, hyperinsulinemia, lipid profile changes, oxidative stress and inflammation contribute to the development of cardiovascular disease beyond non-modifiable factors such as age, gender and genetics 2.

Cardiovascular disease is the main cause of death in dialysis patients 3. The rate of death due to cardiovascular disease reaches 40-50% in patients receiving renal replacement therapies 4. Several studies have showed that peritoneal dialysis patients were more at risk for the development of dyslipidemia and hyperinsulinemia 5,6.

Glucose-based dialysate use causes the absorption of glucose at 100-200 g/day (320-640 kcal/day) in patients receiving peritoneal dialysis. In peritoneal dialysis patients, weight gain occurs in the first year of the therapy, and the use of glucose-based solutions might contribute to this condition 7.

Increased body fat mass, lipid profile disorders and hyperinsulinemia frequently develop in peritoneal dialysis patients, and the risk of these patients developing cardiometabolic syndrome is higher than that of hemodialysis patients 8. For renal replacement therapies, the effects of inflammation and increased oxidative stress on the development of cardiovascular disease have also been shown. Elevated homocysteine levels, high circulating fibrinogen levels, dyslipidemia, an increased body mass index, high C-reactive protein (CRP) levels and malnutrition might increase the risk of cardiovascular disease in dialysis patients. Insulin resistance, and thus hyperinsulinemia, is considered to be another causative factor for cardiovascular disease in end-stage renal disease (ESRD) 3,9.

The present study aimed to compare the cardiovascular risk factors that might be associated with inflammation, atherosclerosis and metabolic syndrome between hemodialysis and peritoneal dialysis patients.

## MATERIALS AND METHODS

Fifty hemodialysis patients (26 males and 24 females) and 50 peritoneal dialysis patients (23 males and 27 females) aged between 18 and 75 years were included in this study. All patients had been receiving dialysis therapy at our center for at least one year without interruption, and their dialysis adequacies were determined. To standardize and homogenize the dialysis therapy groups, the above-mentioned dialysis therapies were managed and followed up by the same team using the same equipment. We excluded patients who had received dialysis therapy at other centers or who had not regularly attended the scheduled follow-up visits during the previous year. Patients with acute infection, chronic liver disease, collagen tissue disease or malignancy were excluded from the study.

The ages and genders of the dialysis patients were recorded. The heights and weights (dry weights) of the patients were determined by using calibrated height/weight scales. The body mass index of each patient was calculated using the formula weight/(height)^2^. The arterial blood pressure of the patients was measured three times on both arms, and the average of the last two measurements was recorded. A desktop sphygmomanometer (Erkameter 3000 with standard mercury) was used to measure the arterial blood pressure. To evaluate the biochemical parameters, venous blood samples were collected after 12 hours of fasting in the morning and before starting dialysis therapy (hemodialysis or peritoneal dialysis). Serum glucose, triglyceride, low-density lipoprotein (LDL)-cholesterol, high-density lipoprotein (HDL)-cholesterol, CRP, fibrinogen and homocysteine levels were measured. Biochemical parameters were measured using an Abbott Aeroset autoanalyzer (Abbott-Aeroset System, Germany). CRP was measured using a Delta SEAC analyzer via the nephelometric method (Radim Reagent, Italy). Fibrinogen testing was performed with a Sysmex CA-1500 analyzer (Dade Behring Thrombin Reagent, Germany) and plasma that was obtained from blood samples in citrated tubes that were centrifuged at 4000 rpm for 15 minutes. To measure the homocysteine levels, blood samples collected in EDTA tubes were centrifuged at 4000 rpm for 15 minutes, without standing time, and the plasma obtained was studied using an Immulite 1000 analyzer (Siemens, USA) via the chemiluminescent method. Carotid Doppler ultrasound of the patients was evaluated using grayscale and color Doppler ultrasonography with a 7.5-mHz linear probe (Toshiba Aplio 80). In a semi-dark room, all subjects lay in the supine position with their necks slightly hyperextended and rotated away from the imaging transducer. The carotid intima media thickness (CIMT) was defined as the distance between the leading edge of the lumen-intima interface and the leading edge of the media-adventitia interface of the far wall. The measurements were taken far from the plaque areas. Three measurements of both carotid arteries were taken proximal to the carotid bifurcation, from which the mean CIMT was derived. Standard bicarbonate dialysates and polysulfone membranes were used in the hemodialysis patients. The patients received hemodialysis therapy three times per week; each session lasted four hours. In the hemodialysis patients, a Kt/V value of 1.4 or more and a urea reduction ratio (URR) value of 70% or more were considered to indicate dialysis adequacy. A standard procedure was performed in the peritoneal dialysis patients (bag changes four times per day, 3x1.36% glucose solutions, 1x3.86% glucose) (Dianeal, Eczacibasi-Baxter, Istanbul, Turkey). A Kt/V value of 1.7 or more was considered adequate in the peritoneal dialysis patients. This study was approved by the ethical committee of the hospital.

### Statistical analysis

To analyze the data obtained in this study, NCSS 2007 & PASS 2008 statistical software (UT, USA) was used. During the evaluation of study data, Student's t test was used for comparisons of parameters between two groups for descriptive statistical measures (mean, standard deviation, and frequency) and for quantitative data. The chi-square test was used to compare qualitative data. The results were evaluated within a 95% confidence interval and at a significance level of *p<*0.05. The Mann-Whitney U test was used to compare parameters without a normal distribution between two groups. The results were evaluated within a 95% confidence interval and at a significance level of *p<*0.05.

## RESULTS

A total of 100 patients (50 hemodialysis and 50 peritoneal dialysis patients) who were regularly followed up for more than one year were included in this study. Table 1 shows the demographic characteristics of the patient groups (Table 1). No significant difference was found between the hemodialysis and the peritoneal dialysis patients in terms of age (*p=*0.269), gender (*p=*0.683), body mass index (*p=*0.426), dialysis duration (*p=*0.052), systolic blood pressure (*p=*0.160) or diastolic blood pressure (*p=*0.387) (Table 1). No significant difference was found between the hemodialysis group and the peritoneal dialysis group regarding the proportion of patients with diabetes mellitus (10/50 versus 7/50, *p>*0.05)

Fasting blood glucose (*p=*0.006), LDL-cholesterol (*p=*0.001), triglyceride (*p=*0.001), HDL-cholesterol (*p=*0.0001), fibrinogen (*p=*0.001) and homocysteine (*p=*0.002) levels were significantly higher in the peritoneal dialysis patient group compared with the hemodialysis patient group (Table 2). No significant differences in triglyceride (*p=*0.708), HDL-cholesterol (*p=*0.631), CRP (*p=*0.828) or homocysteine (*p=*0.012) levels were found between the patients with diabetes mellitus and without diabetes mellitus who were undergoing renal replacement therapy via either modality (*p>*0.05) (Table 3). The mean fasting blood glucose (*p=*0.001), LDL-cholesterol (*p=*0.001) and fibrinogen (*p=*0.0049) levels of the patients with diabetes mellitus (n=17) who were undergoing peritoneal dialysis or hemodialysis were significantly higher than those of patients without diabetes mellitus who were undergoing peritoneal dialysis or hemodialysis (*p<*0.05) (Table 3).

## DISCUSSION

Traditional risk factors and ESRD-related risk factors contribute to the high prevalence of cardiovascular disease in dialysis patients 10. Atherosclerosis and chronic inflammation are strongly associated with cardiovascular disease in patients undergoing long-term dialysis therapy 2. Hemodialysis and peritoneal dialysis are generally associated with similar long-term survival 11. In studies related to life expectancy and quality of life, cardiovascular disease risk was the focus of investigation. The importance of cardiometabolic syndrome and its effects on cardiovascular disease is a current issue 12. To investigate the risk of cardiovascular disease in dialysis patients, factors including obesity, lipid profiles, insulin resistance and markers of inflammation were compared in dialysis patients by Kam-Too et al. The frequency of metabolic syndrome was approximately 40% in hemodialysis patients and approximately 60% in peritoneal dialysis patients. The authors reported that the frequency of cardiovascular disease might be higher in patients with metabolic syndrome 13.

McIntyre et al. noted that the glucose-based solutions used in peritoneal dialysis could significant contribute to the development of obesity as well as metabolic syndrome in peritoneal dialysis patients compared with hemodialysis patients. Glucose-based solutions used intraperitoneally to treat peritoneal dialysis patients also had systemic glucotoxic effects beyond local peritoneal effects. Given that functional and structural changes occurred in peritoneal membranes with the use of glucose-based solutions, an elevated advanced glycation end-product concentration in the skin of diabetic and non-diabetic peritoneal dialysis patients parallel to dialysis vintage was shown to be evidence of systemic glucotoxicity 14.

According to the studies performed regarding the frequency and significance of cardiometabolic syndrome in peritoneal dialysis patients, dyslipidemia develops in addition to weight gain in most patients, especially within the first year 13,15.

In our study, no difference between the dialysis patient groups regarding body mass index was observed, but fasting blood glucose, LDL-cholesterol, triglyceride and HDL-cholesterol levels were significantly higher in peritoneal dialysis patients. No significant difference in the rate of diabetes mellitus was found between the study groups. We suggested that the higher levels of blood glucose and LDL-cholesterol in the peritoneal dialysis patients in our study group might have been due to glucotoxic effects caused by the glucose-based solutions used and that these levels may indicate an increased risk of atherosclerosis. These results were in agreement with those of other studies reporting similar results 5,7. Huang et al. demonstrated that LDL-cholesterol and apolipoprotein B levels were higher in peritoneal dialysis patients and concluded that atherosclerosis might be more prevalent in these patients 15. The reduction of LDL-cholesterol and lipoprotein levels in patients switched from peritoneal dialysis to hemodialysis also supported the concept that dyslipidemia, and thus atherosclerosis, may occur more commonly in peritoneal dialysis patients 16.

In our study, to determine the presence of inflammation and atherosclerosis in peritoneal dialysis and hemodialysis patients, we examined homocysteine and fibrinogen levels and the presence of atherosclerotic plaques in the carotid artery. In the peritoneal dialysis patient group, plasma fibrinogen and serum homocysteine levels were significantly higher, but no difference was found between the two dialysis groups regarding the presence of atherosclerotic plaques in the carotid artery.

Several studies have shown the presence of chronic inflammation in every stage of chronic renal disease, with inflammation increasing with disease progression 17. Plasma fibrinogen levels were increased in the presence of inflammation, and these levels were an important risk factor for the development of cardiovascular disease. In the current study, fibrinogen levels were increased with both types of dialysis therapies. However, different results were obtained when comparing fibrinogen levels between the hemodialysis and peritoneal dialysis therapies. The results of certain studies have shown that fibrinogen levels were higher in peritoneal dialysis patients when the serum cholesterol and lipoprotein (a) levels were similar to those in hemodialysis patients and that this condition was associated with a higher risk of developing atherosclerosis in peritoneal dialysis therapy 18.

The reason for elevated fibrinogen levels in peritoneal dialysis patients remains unclear, but factors such as peritoneal protein loss and hyperinsulinemia might cause the increase in this patient group 18,19. Homocysteine levels are also increased in patients with moderately impaired renal function. The majority of chronic renal disease patients have elevated homocysteine levels that are 3- to 4-fold above normal 17. Hyperhomocysteinemia is considered to be an important risk factor in the development of atherosclerotic vascular disease 20. Homocysteine levels are higher in peritoneal dialysis patients, independent of age, dialysis adequacy, serum B_6_ levels, and protein catabolic rates 21. In our study, the elevated homocysteine levels in peritoneal dialysis patients may support the concept that peritoneal dialysis patients are at a greater risk for atherosclerosis. Peritoneal dialysis solutions that cannot remove homocysteine from the body might also have contributed to the elevated homocysteine levels. However, considering that the effects of the hemodialysis and peritoneal dialysis modalities on serum homocysteine levels are generally similar, higher serum homocysteine levels in peritoneal dialysis patients might be associated with an increased presence of atherosclerosis.

An increased CIMT and the presence of atherosclerotic plaques are generally considered to be indicators of subclinical atherosclerosis. Several studies have shown that increased levels of LDL and the atherogenic index of plasma are related to an increased CIMT and the presence of atherosclerotic plaques. In particular, the atherogenic index of plasma and the CIMT were increased in dialysis patients compared with a healthy control group (1). In our study, the frequency of atherosclerotic plaques was higher in peritoneal dialysis patients, but the difference was not significant (*p>*0.05). Thus, we could not evaluate our study parameters by regression analysis methods.

Our study was a cross-sectional observational study, so we could not evaluate the cardiovascular complications and/or mortality rates of our dialysis patients. However, a review of the data from our dialysis clinic over the past year showed that only 1 hemodialysis patient (and no peritoneal dialysis patients) had died. Additionally, three peritoneal dialysis patients and one hemodialysis patient underwent kidney transplantation during this period (unpublished data).

Traditional risk factors and ESRD-related risk factors contribute to the high prevalence of cardiovascular disease in dialysis patients. Atherosclerosis and chronic inflammation are strongly associated with cardiovascular disease in patients undergoing long-term dialysis therapy. Most studies have shown that peritoneal dialysis patients are at greater risk for cardiovascular disease and atherosclerosis. In our study, peritoneal dialysis patients had a significant increase in cardiovascular risk factors but a non-significant increase in subclinical atherosclerosis. Further studies are required in this field.

## Figures and Tables

**Table 1:** -Demographic Characteristics of Hemodialysis and Peritoneal Dialysis Patients.

Parameter	Hemodialysis Patients (n=50)	Peritoneal Dialysis Patients (n=50)	*p*-value
+**Age (years)**	43.00±13.00	46.00±14.00	**0.269**
++**Gender** ** Female** ** Male**	48.00% (n=24)52.00% (n=26)	54.00% (n=27)46.00% (n=23)	**0.683**
+**Body mass index (kg/m^2^)**	24.42±1.56	23.85±2.75	**0.426**
+**Systolic blood pressure (mm/Hg)**	111±13.72	120±24.49	**0.160**
+**Diastolic blood pressure (mm/Hg)**	73±13.02	76.50±12.25	**0.387**
+**Dialysis duration (months)**	24.00±12.00	20.00±8.00	**0.052**

+ Student's t test.

++ Chi-square test.

**Table 2:** -Comparison of Clinical and Biochemical Parameters between Hemodialysis and Peritoneal Dialysis Patients.

Parameter	Hemodialysis Patients	Peritoneal Dialysis Patients	*p*-value
+**Fasting blood glucose (mg/dl)**	108.00±48.00	136.00±52.00	**0.006**
+**LDL-cholesterol (mg/dl)**	91.00±22.30	123.00±37.30	**0.001**
+**Triglyceride (mg/dl)**	127.20±18.50	167.40±24.70	**0.001**
+**HDL-cholesterol (mg/dl)**	41.30±5.70	33.60±7.90	**0.001**
+**C-reactive protein (mg/dl)**	2.16±1.87	2.91±2.00	**0.229**
+**Fibrinogen (IU/ml)**	282.53±95.78	402.20±105.21	**0.001**
+**Homocysteine (IU/ml)**	10.82±3.74	20.20±11.37	**0.002**
++**Presence of atherosclerotic plaque in carotid artery**	24.00% (n=12)	44.00% (n=22)	**0.185**

+ Student’s t test.

++ Chi-square test.

**Table 3:** -Biochemical Data of Diabetes Mellitus and Non-Diabetes Mellitus Dialysis Patient Groups.

**Biochemical Parameter**	**Diabetes Mellitus (n=17)**	**Non-Diabetes Mellitus (n=83)**	*p***-value**
**Fasting blood glucose (mg/dl)**	162.90±36.12	96.39±16.89	0.001
**LDL-cholesterol (mg/dl)**	131.80±55.91	91.63±28.84	0.001
**Triglyceride (mg/dl)**	144.34±61.84	167.36±108.57	0.708
**HDL-cholesterol (mg/dl)**	41.26±12.11	39.90±9.52	0.631
**CRP (mg/dl)**	2.75±1.91	2.82±2.28	0.828
**Homocysteine (IU/ml)**	18.90±10.39	13.76±8.74	0.012
**Fibrinogen (IU/ml)**	395.15±107.67	308.52±107.30	0.004

Mann-Whitney U test: *p<*0.05.
